# Impact of Dehydration Techniques on the Nutritional and Microbial Profiles of Dried Mushrooms

**DOI:** 10.3390/foods13203245

**Published:** 2024-10-12

**Authors:** Imane Moutia, Erika Lakatos, Attila József Kovács

**Affiliations:** 1 Department of Biosystems Engineering and Precision Technology, Albert Kázmér Mosonmagyaróvár Faculty of Agricultural and Food Sciences, Széchenyi István University, Vár tér 2, H-9200 Mosonmagyaróvár, Hungary; moutia.imane@ga.sze.hu; 2 Department of Food Science, Albert Kázmér Mosonmagyaróvár Faculty of Agricultural and Food Sciences, Széchenyi István University, Lucsony utca 15-17, H-9200 Mosonmagyaróvár, Hungary; lakatos.erika@sze.hu

**Keywords:** food preservation, dehydration effects, microbial safety in dried mushrooms, dehydration methods and bioactive compounds, mushroom dehydration techniques

## Abstract

The global consumption of dried mushrooms has increased worldwide because of their rich nutritional value and culinary versatility. Dehydration methods such as sun drying, hot air drying, freeze drying, and microwave drying are employed to prolong the shelf life of a food product. These methods can also affect the food product’s nutritional value and the final product’s microbial profile. Each technique affects the retention of essential nutrients like vitamins, minerals, and bioactive compounds differently. Additionally, these techniques vary in their effectiveness at reducing microbial load, impacting the dried mushrooms’ safety and shelf life. This review addresses the gap in understanding how different dehydration methods influence dried mushrooms’ nutritional quality and microbial safety, which is crucial for optimizing their processing and consumption. It targets researchers, food processors, and consumers seeking to improve the quality and safety of dried mushrooms. This review comprehensively examines the impact of major dehydration techniques, including sun drying, hot air drying, microwave drying, and freeze drying, on the nutritional and microbial profiles of dried mushrooms. Each method is evaluated for its effectiveness in preserving essential nutrients and reducing microbial load. Current research indicates that freeze drying is particularly effective in preserving nutritional quality, while hot air and microwave drying significantly reduce microbial load. However, more well-designed studies are needed to fully understand the implications of these methods for safety and nutritional benefits. These findings are valuable for optimizing dehydration methods for high-quality dried mushrooms that are suited for culinary and medicinal use.

## 1. Introduction

Mushrooms are valued worldwide for their rich nutritional content and potential medicinal properties. They provide essential nutrients, including vitamins, minerals, proteins, and carbohydrates, making them a valuable component of various diets and for maintaining overall health [1,2,3]. Mushroom production in Europe has steadily grown in recent years, contributing substantially to the agricultural sector. According to recent statistics, the European Union (EU) is a major producer of mushrooms, with over 3 million tonnes being produced annually [4]. Mushroom production has experienced a consistent rise on a global scale, with numerous countries emerging as key contributors to the international market. The industry predominantly centers on the cultivation of button mushrooms (*Agaricus bisporus*) and oyster mushrooms (*Pleurotus eryngii*). In recent years, annual mushroom production has reached impressive figures, playing a crucial role in both domestic consumption and export activities. Approximately 75% of button mushrooms are marketed fresh, while the remaining 25% are processed into their canned or dried forms. In addition to fresh exports, various nations also engage in the export of dried mushrooms, including shiitake (*Lentinula edodes*). Moreover, the dried mushroom segment has witnessed substantial growth, driven by an increasing demand for products that offer extended shelf life and enhanced flavor concentration. This growth is evident in the rising export figures, with dried mushroom exports representing a significant portion of the overall mushroom market. The development of both fresh and dried mushroom sectors underscores the industry’s vital role in agriculture and its potential for continued expansion within the global marketplace [4,5].

Numerous studies have consistently demonstrated that dried mushrooms serve as a rich source of vitamin D. Research has shown that the drying process, particularly when mushrooms are exposed to sunlight or ultraviolet (UV) radiation, significantly enhances their vitamin D content [6,7]. However, the high moisture content of mushrooms and their perishable nature require effective preservation methods to extend their shelf life and retain their nutritional benefits. Dehydration is a widely used method for preserving mushrooms, which involves removing water to inhibit microbial growth and enzymatic activity [2].

The significance of dehydrated mushrooms extends beyond their culinary use, as they are also used in traditional and modern medicinal practices. Ensuring the nutritional integrity and microbial safety of dried mushrooms is mandatory, as these factors directly influence their quality and health benefits. Dehydration techniques are fundamental in food processing, serving to significantly reduce the moisture content of food products in order to extend their shelf life, maintain nutritional value, and preserve sensory qualities such as texture, color, and flavor. In addition to the drying method, pretreatments may help reduce the drying time and improve the moisture diffusion coefficient in the mushrooms during dehydration [8].

Among these methods, convection drying is widely used, whereby heat is transferred to the food via fluid (air or gas) movement. This category encompasses techniques such as hot air drying, which is commonly used for fruits, vegetables, and mushrooms; spray drying, which is particularly suitable for liquid or semi-liquid food products like milk and juices; and fluidized bed drying, which efficiently handles particulate food materials by suspending them in a hot air stream, enhancing heat and mass transfer. To improve the drying process, pretreatments such as blanching—brief exposure to boiling water or steam to inactivate any enzymes—and osmotic dehydration—immersion in a concentrated solution to remove water—are often applied to improve the texture, flavor, and drying rate [9,10,11,12]. Conduction drying, on the other hand, is characterized by direct contact between the food and a heated surface, making it particularly energy efficient. Techniques such as tray drying and vacuum drying fall under this category. Tray drying is ideal for thin layers of food, while vacuum drying, which is often paired with freezing or preheating, is employed for heat-sensitive products like fruits and vegetables to retain their quality [13,14]. Radiation drying, including the use of infrared and microwave energy, utilizes electromagnetic waves to remove moisture quickly and efficiently. This method is well-suited for products where rapid drying is necessary to prevent quality loss. For instance, infrared drying penetrates the food surface to accelerate drying, while microwave drying allows for volumetric heating, ensuring uniform moisture removal from the product’s interior. Pretreatments such as chemical treatments to inhibit enzymatic browning or blanching to soften the food matrix are commonly paired with radiation drying to maintain color, texture, and nutrient retention [9,13,15].

Despite the extensive use of dehydration techniques, significant gaps remain in the available research regarding their comparative effectiveness in preserving essential nutrients and ensuring microbial safety. This review aims to fill these gaps by evaluating the effects of different widely used dehydration methods on dried mushrooms. By synthesizing the current research findings, we seek to identify the most effective dehydration techniques that balance preservation efficacy with the retention of nutritional integrity and microbial safety. This review is structured to provide a comprehensive analysis of each dehydration method, followed by a discussion on nutritional and microbial considerations. The insights gained from this review will be valuable for researchers, the food-processing industry, and consumers seeking to enhance the quality of dried mushrooms for both culinary and medicinal purposes.

## 2. Overview of Mushrooms and Their Nutritional Value

Various research articles provide a comprehensive and detailed overview of the numerous types of mushrooms and their extensive therapeutic potential. Mushrooms are rich in bioactive compounds, including polysaccharides, terpenoids, phenolic compounds, and essential nutrients such as proteins, vitamins, and minerals. These components collectively contribute to the immense health benefits associated with mushrooms, making them invaluable for human health [16,17]. With a protein content ranging from 20 to 40% and some species achieving up to 30% protein by dry weight, mushrooms surpass many vegetables, fruits, and grains as an alternative protein source [18,19]. Mushroom proteins are linked to various health-promoting properties, including angiotensin-converting enzyme (ACE) inhibition and antioxidant, antimicrobial, anticancer, and gut microbiota modulation effects [20]. Additionally, mushrooms are rich in vitamins such as vitamin D, B2, and B12, and minerals like copper, selenium, and potassium, enhancing their role in improving human health [18,19].

Edible mushrooms such as oyster mushrooms (*Pleurotus ostreatus*), reishi mushrooms (*Ganoderma lucidum*), maitake mushrooms (*Grifola frondose*), and shiitake mushrooms (*Lentinus edodes*) have been extensively studied for their notable anti-diabetic and cholesterol-reducing properties [18]. Research indicates that these mushrooms can help regulate blood sugar levels and reduce cholesterol, thus supporting cardiovascular health. Moreover, certain mushrooms, including species from the *Psilocybe* genus, contain potent psychoactive components such as psilocybin and psilocin. These compounds have shown significant therapeutic potential in the treatment of mental health disorders, including depression, anxiety, and post-traumatic stress disorder (PTSD). Clinical trials and studies have demonstrated that psilocybin, in particular, can lead to profound and lasting improvements in mental well-being when used under controlled conditions [16,21].

## 3. Dehydration Methods for Mushroom Preservation

Dehydration is the process of removing moisture or water content from food products to preserve them for longer periods. By reducing the water activity in food, microbial growth, enzymatic reactions, and chemical changes are inhibited, thus preventing spoilage [22]. This process can be achieved through various methods such as hot air drying, freeze drying, or sun drying, depending on the food product and the desired characteristics. Dehydrated foods typically have a longer shelf life, reduced weight for easier transportation, and sometimes concentrated flavors. However, the process of dehydration can also affect the texture, color, and nutritional content of the food [23].

Various studies have explored different dehydration methods and pretreatments to optimize the dehydration process for mushrooms, including oven drying, sun drying, and cabinet drying, with pretreatments like blanching and sulfiting [24,25]. Bora and Kawatra highlighted the effectiveness of dehydration in extending the shelf life of oyster mushrooms (*Pleurotus florida*), noting that different pretreatments affect their rehydration properties. Similarly, Stavropoulou et al. emphasized the benefits of osmotic dehydration in preserving the quality of button mushrooms (*Agaricus bisporus*)*,* especially when followed by freezing [26]. These studies collectively underscore the importance of selecting appropriate dehydration techniques and pretreatments to enhance the shelf life, nutrient retention, and overall quality of dehydrated mushrooms for various applications.

According to the literature, relevant dehydration methods for mushrooms specifically include hot air drying, freeze drying, and microwave drying for mushrooms, as well as sun drying, which, despite its negative effects, is one of the oldest methods used [27,28].

### 3.1. Sun Drying

Sun drying is a common and cost-effective method for preserving mushrooms by exposing them to direct sunlight. However, the low temperatures involved in this process can compromise the quality of the mushrooms, resulting in an unpleasant appearance, aroma, and flavor compared to mushrooms dried using higher-temperature methods like hot air drying. Research on oyster mushrooms (*Pleurotus ostreatus*) has shown that while sun drying is effective, it can lead to a darker color compared to other drying methods [29]. Despite this, sun-dried oyster mushrooms (*Pleurotus ostreatus*) still meet product standards in terms of color, size, odor, cleanliness, and moisture content. To optimize the quality and efficiency of sun drying, it is crucial to control factors such as temperature and relative humidity [30]. Additionally, studies have indicated that sun drying can increase the vitamin D content of mushrooms by exposing these food commodities to UV-B radiation. Multiple studies confirm that exposing mushrooms to UV-B radiation, whether from sunlight during sun drying or from artificial sources, can significantly boost their vitamin D2 content, enhancing their nutritional profile. The extent of this increase depends on factors such as mushroom type and UV dose [31,32].

Research has explored various drying methods, including solar drying, to preserve mushrooms effectively. Studies have shown that using solar dryers with specific structures can enhance the drying process, resulting in reduced drying times compared to open sun drying. Furthermore, solar drying systems provide a more controlled environment, protecting the mushrooms from external damage [33]. The application of different pretreatment conditions, such as sulfiting, has also been found to improve the dehydration characteristics of mushrooms during drying, leading to better nutrient retention and better overall product quality [18]. Additionally, inflatable solar dryers have been shown to significantly reduce the moisture content of mushrooms within a few hours, maintaining product quality while being economically feasible [34]. These studies highlight the importance of efficient drying techniques, such as solar drying, in preserving mushrooms while maintaining their nutritional value and quality.

Overall, although sun drying has its drawbacks, it can be a simple and cost-effective method for drying mushrooms when high quality is not a strict requirement [29,35].

### 3.2. Convection Drying

In convection drying, heat is transferred directly to the material by hot air, raising its temperature and promoting moisture evaporation. As moisture evaporates from the material’s surface, it increases the humidity of the surrounding air. This humid air is continuously removed or replaced with drier air to maintain an efficient drying process, enhancing both heat and mass transfer. Convection drying methods, including hot air drying, tray drying, and tunnel drying, are widely used as dehydration methods [36,37]. The use of forced convection methods can notably shorten drying times compared to natural convection methods [38]. For instance, in typical dehydrators, mushrooms are dried for 8 to 10 h, whereas conventional ovens, which rely on natural convection and radiation for heat transfer, may require twice as long.

Convection drying efficiency is influenced by several critical parameters, including particle size, temperature, and humidity, which collectively determine the drying rate and moisture content. The particle size of mushroom slices, for instance, significantly affects both the efficiency and quality of drying processes. Research indicates that thinner slices of 2.5 mm yield better rehydration ratios and color retention compared to thicker slices of 5 mm, which exhibit lower effective diffusivity coefficients and slower drying rates [39,40].

Controlling the drying temperature is crucial for optimal results. The air temperature at the inlet of the drying chamber typically ranges from 55 °C to 60 °C, while the outlet temperature can vary, with studies indicating a maximum temperature difference of about 4 °C between the inlet and outlet [41]. In solar drying systems, the average air temperature can reach around 55 °C under optimal conditions, with product temperatures generally remaining below 40 °C [42]. Research indicates that temperatures of up to 65 °C have been used. The optimal range for convection-based mushroom drying is between 50 °C and 60 °C, with 50 °C frequently being identified as the ideal temperature in multiple studies. At these temperatures, drying times generally span from 8 to 12 h [7,43]. However, convection drying processes may compromise the quality and nutritional content of the final product, due to prolonged exposure to elevated temperatures [36].

The role of air humidity in the drying process is critical, as lower humidity levels significantly enhance the drying rates [44]. Typically, the relative humidity of drying air ranges from 20% to 70%, with optimal drying efficiency observed at lower humidity levels [45]. The best rehydration results for oyster mushrooms (*Pleurotus ostreatus*) were achieved at a lower drying temperature of 40 °C and a higher relative humidity of 75% during dehydration [46].

Convection drying methods are essential for preserving mushrooms while maintaining their quality and nutritional value [22,47]. These methods, particularly hot air drying, have demonstrated that they bring enhancements in taste, nutritional attributes, and shelf life [48]. Research emphasizes the efficacy of drying in removing moisture content, deactivating enzymes, and preventing microbial proliferation [43]. Furthermore, studies highlight how forced convection drying significantly reduces the drying times and impacts mushroom coloration. Using the optimal drying parameters and pretreatments, such as blanching, is crucial for improving drying efficiency and ensuring high-quality dried products. Comparative research indicates that convection methods may be less energy efficient and require longer drying times than advanced techniques like microwave-assisted convective drying [49]. Overall, convection drying methods remain indispensable for efficiently preserving mushrooms while preserving their sensory qualities and nutritional integrity.

### 3.3. Microwave Drying

The microwave drying of mushrooms is a method that aims to reduce moisture content efficiently while preserving quality attributes. Studies have shown that microwave power levels and pretreatments like blanching significantly impact drying characteristics [50,51]. Microwave drying is an effective method for preserving mushrooms, with little deterioration in flavor or quality. Studies have shown that microwave-assisted hot air drying can significantly reduce mushroom drying time compared to using hot air drying only [27,52,53].

Combining microwave with convection drying can lead to shorter drying times, higher diffusion rates, and better energy efficiency compared to conventional methods [49]. Additionally, tempering treatments during microwave vacuum drying can enhance product quality by improving effective moisture diffusivity and reducing drying times [54]. Furthermore, combining airborne ultrasound methods with microwave vacuum drying results in a porous structure that helps maintain nutrient levels and quality attributes in dehydrated mushrooms [55]. These findings collectively highlight the potential of microwave drying techniques in producing high-quality dried mushrooms efficiently.

Another study investigated how microwave power, air temperature, and air velocity impact the drying process of sliced mushrooms. The results showed that increasing the microwave power and air velocity significantly reduced the drying time, whereas higher air temperatures had a lesser effect [53]. Optimal drying occurred at air temperatures of around 40 and 80 °C and air velocities of 0.5–1.5 m/s [27,53]. The effective temperature for microwave drying typically ranges from 60 °C to 80 °C, depending on the specific mushroom type and desired moisture content [43,56].

Microwave drying of mushrooms involves specific power levels that significantly influence the drying kinetics and quality of the final product. Research indicates that microwave power densities for the effective drying of mushrooms typically range from 20 to 60 W/g [40]. Moderate power levels, combined with temperature control, yield dehydrated mushrooms of a quality comparable to freeze-dried samples, indicating that higher microwave power does not always equate to better quality [57]. During a study, the drying trials were conducted at microwave power densities between 2.4 W/g and 14 W/g. The product temperatures during drying can vary significantly based on these power settings, with higher power leading to quicker moisture removal and potentially higher product temperatures [58].

In summary, microwave-assisted hot-air drying can dry mushrooms to the desired 10% moisture content in almost 5 min, this being significantly faster than hot-air drying alone, which takes over 2.5 h [27,52,53]. Specifically, one study found that mushrooms could be microwave and hot-air dried down to 10% moisture content in just 5 min, a four-fold reduction in drying time compared to hot-air drying at 80 °C, which took 170 min. The microwave drying process involved a short initial exposure to microwave energy for around 5% of the total drying time, followed by longer convective drying. This combination allowed the mushrooms to be dried quickly while maintaining high quality, with the samples exhibiting a uniform beige-golden color and the decreased size and wrinkling characteristics of properly dried mushrooms [27].

### 3.4. Freeze Drying

Freeze drying is a highly advantageous method for preserving mushrooms, as it effectively retains their bioactive compounds and nutritional qualities. The freeze-drying process involves complex heat transport mechanisms, wherein heat is primarily transferred to the product through a shelf, utilizing temperature-controlled fluids. This method is essential during the primary drying phase, where sublimation occurs at low temperatures (typically −40 °C to −10 °C) [59]. This technique involves freezing the product and then sublimating the ice under vacuum conditions, which offers several advantages over conventional drying methods. Freeze drying takes between 12 and 24 h to complete the drying process, depending on factors such as mushroom size and initial moisture content [28]. In freeze drying, the air temperature during the drying process does not exceed 25 °C [2]. Initially, the product is frozen to a temperature typically below −40 °C. Mushrooms are typically frozen at temperatures around −80 °C [60].

Maintaining a low-pressure vacuum environment is crucial during this process to facilitate sublimation, prevent enzymatic degradation, and maintain the mushrooms’ natural color [60]. Studies have consistently shown that freeze drying preserves the mushrooms’ texture, color, flavor, and nutritional content more effectively than alternatives, such as hot air drying. Although freeze drying is recognized as the most expensive drying method, due to the need for specialized equipment and extended processing times, it is effective in preserving the quality of mushrooms. This technique maintains the product’s original characteristics, including color, texture, flavor, and nutritional value. In comparison to other drying methods, such as hot air drying, which can alter texture and cause color loss, freeze drying effectively ensures that the mushrooms retain their attractive appearance and overall integrity, making it a preferred choice for high-quality culinary and nutritional applications [28,60,61].

Previous research supports freeze drying as a superior choice for preserving mushrooms’ quality attributes [61]. Studies have demonstrated that freeze-dried mushrooms exhibit lower moisture content and water activity than those dried in cabinet settings [60]. Alongside this, they also present higher levels of crude protein, crude fiber, crude fat, ash, carbohydrates, and energy content [22,60]. Moreover, freeze drying better maintains certain compounds such as total phenolic content compared to other drying methods [62]. The scientific literature consistently highlights the finding that freeze drying yields dried mushrooms with superior texture and overall quality compared to other drying techniques. However, it is noted that this method requires longer drying times and incurs higher costs.

In conclusion, freeze drying emerges as a preferred technique for preserving mushrooms due to its ability to maintain their nutritional profile, bioactive compounds, and desirable sensory attributes. Previous studies consistently underline its effectiveness in achieving better color parameters and overall quality compared to alternative drying methods.

### 3.5. Combined Dehydration Methods

Combined dehydration methods integrate multiple techniques to optimize moisture removal while preserving the sensory and nutritional attributes of food products. These methods can be categorized into two types. The first type, known as serial combined drying, entails the sequential application of different drying techniques, such as employing an instant controlled pressure drop (DIC) technique followed by hot air drying, or freeze drying (FD) followed by hot air drying. This approach has demonstrated significant enhancements in rehydration quality, with DIC treatment preceding hot air drying at 35 °C proving particularly effective. This sequence improves the porous structure of mushrooms, resulting in greater volume recovery and reduced dry matter loss during rehydration.

The second type, referred to as parallel combined drying, involves the simultaneous implementation of two or more drying methods. This approach necessitates specialized equipment and a higher financial investment, rendering it less accessible than serial methods [63].

Combined dehydration methods, particularly when used to dry mushrooms, enhance drying efficiency and product quality. Notably, freeze drying combined with microwave vacuum drying has shown significant advantages in terms of drying kinetics and rehydration characteristics for white button mushrooms (*Agaricus bisporus*). The effective diffusivity during the microwave vacuum-drying phase was found to be ten times greater than with freeze drying alone, leading to faster moisture removal [40]. Osmotic dehydration is often used as a pretreatment before conventional drying methods like hot air drying or freeze drying. It reduces moisture content significantly and modifies the physical properties of mushrooms, leading to shorter drying times and energy savings. For instance, osmotic dehydration with oligofructose or maltodextrin can enhance the texture and color retention of dried mushroom products [25,64]. Studies indicate that combining osmotic dehydration with hot air drying results in a significant reduction in drying time compared to untreated mushrooms, while also extending shelf life [64,65].

The integration of various dehydration techniques presents a promising approach for improving the quality and efficiency of mushroom preservation. These methods not only enhance the product’s rehydration properties but also contribute to energy savings and extended shelf life, making them ideal for commercial applications in the food industry.

## 4. Nutritional Impact of Dehydration Techniques

Various dehydration techniques can have different impacts on the nutritional quality of the final product. This chapter examines the effect of common dehydration methods, including air drying, freeze drying, and sun drying, on the nutritional composition of food. The main nutritional content variations in different edible mushroom species using different drying techniques are summarized in Table 1.

The table titled “Effects of various drying techniques on the nutritional composition of different mushroom species” provides a comparative analysis of how different dehydration methods, including sun drying, hot air drying, microwave drying, and freeze drying, affect the nutritional composition of different mushroom species. The table includes data on key nutrients such as vitamin D content and bioactive compounds. The table highlights the variations in nutrient retention across the different drying methods, offering insights into the most effective techniques for preserving the nutritional quality of each mushroom species.

### 4.1. Proteins

Fresh and dried mushrooms are both excellent sources of high-quality protein, with significant variations based on species and drying methods. Fresh mushrooms generally have a high protein content, with an average of 23.80 g/100 g. Popular species like shiitake mushrooms (*Lentinus edodes*), white button mushrooms (*Agaricus* spp.), and oyster mushrooms (*Pleurotus* spp.) contain 14.87–27.13 g/100 g, 26.60–39.84 g/100 g, and 18–19.15 g/100 g of protein content, respectively. Indeed, the protein content of mushrooms varies not only by species but also by geographical position. Different species of mushrooms naturally have varying levels of protein, and environmental factors such as soil composition, climate, and altitude can further influence the nutritional content of mushrooms grown in different regions. In the Kilum-Ijim forest of Cameroon, the crude protein content in different mushroom species ranges from 6.60 to 30.69 g/100 g. For instance, black forest mushroom (*Lentinus squarrosulus*) powder has an exceptional protein value of 30.12 g/100 g on a dry weight basis. Furthermore, protein content can also vary within different parts of the same mushroom; for instance, the fringed sawgill (*Lentinus crinitus*) has 14.4 g/100 g protein in the pileus and 9.5 g/100 g in the stipe [20].

Research on protein content in fresh and dried mushrooms with various drying methods reveals significant findings. Adhikari et al. found that sulfited samples showed the best retention of crude protein during the drying process, with values ranging from 21.91 g/100 g to 23.17 g/100 g on a dry weight basis [18]. Priyadarsini et al. observed that hot air drying at 40 °C resulted in the highest protein content in oyster mushrooms (*Pleurotus ostreatus*) at 30.8 g/100 g on a dry weight basis [68]. In a recent study by Adhikari et al. in 2022, examining the dehydration properties of milky white mushrooms (*Calocybe indica*) under different pretreatment conditions and drying mechanisms, the crude protein content of fresh untreated samples was analyzed across various drying methods. The results indicated that cabinet drying preserved the highest crude protein content at 22.85 g/100 g on a dry weight basis, followed by solar drying at 21.52 g/100 g, and sun drying at 20.73 g/100 g [76]. Freeze drying preserves the protein content effectively, with studies showing that it can enhance protein levels significantly, reaching up to 24.36 g/100 g on a dry weight basis in some cases [77].

Microwave drying, while faster than freeze drying, tends to result in a greater loss of nutrients, especially proteins. The rapid heating process inherent in microwave drying can cause structural changes in proteins, resulting in denaturation and a reduction in their functional properties. Although there have been improvements in the microwave-assisted drying methods designed to better preserve the nutrients, these techniques generally yield a lower protein content relative to freeze drying [78,79].

These studies collectively demonstrate that different drying methods can impact the protein content of mushrooms, with variations depending on the specific drying technique employed.

### 4.2. Carbohydrates

Mushrooms are generally low in carbohydrates, making them suitable for those following a low-carbohydrate or keto diet. The carbohydrate content of fresh mushrooms ranges from 2.5 to 4 g/100 g 4 g/100 g, with variations in carbohydrate content observed after drying [80,81]. Drying mushrooms concentrates their carbohydrate content. For instance, air-dried white button mushrooms (*Agaricus bisporus*) contain 8.1 g/100 g of total carbs on a dry weight basis, while freeze-dried samples contain 10.1 g/100 g on a dry weight basis. Different drying methods for shiitake mushrooms (*Lentinula edodes*) have a carbohydrate content ranging from 7.6 g/100 g to 11.4 g/100 g on a dry weight basis. Thus, dried mushrooms generally have higher carbohydrate counts on a dry weight basis than fresh mushrooms [69]. In summary, while fresh mushrooms are keto-friendly, due to their low carbohydrate content, the increased carbohydrate levels in dried mushrooms necessitate careful consideration when they are included in such diets.

As reported by Adhikari et al., the carbohydrate content of fresh *Calocybe indica* (milky white mushrooms) was analyzed under various drying conditions to determine the effect of different pretreatment and drying methods on their nutritional composition. For fresh samples, the cabinet drying method resulted in a carbohydrate content of 51.24% on a dry weight basis, solar drying yielded a slightly higher carbohydrate content of 52.56%, and sun drying provided the highest carbohydrate content at 54.33% on a dry weight basis [18]. These variations suggest that sun drying may enhance carbohydrate retention in mushrooms, potentially due to the lower drying temperatures preserving more carbohydrates compared to controlled cabinet drying or solar drying. Understanding these differences is crucial for optimizing drying techniques to maintain or enhance the nutritional value of mushrooms in food processing.

### 4.3. Fibers

Mushrooms are a rich source of dietary fiber, with total dietary fiber (TDF) contents ranging from 11 to 64 g/100 g on a dry weight basis. The majority of this fiber is insoluble dietary fiber (IDF), comprising 12–21 g/100 g of TDF in wild mushrooms and 30.7–42.4 g/100 g on a dry weight basis in oyster mushrooms (*Pleurotus* Spp.), while the soluble dietary fiber (SDF) typically accounts for 2–12 g/100 g on a dry weight basis. Specifically, oyster mushrooms (*Pleurotus ostreatus*) contain 47.3 g/100 g TDF, with 42.4 g/100 g IDF and 5.0 g/100 g SDF, and *Pleurotus eryngii* (king oyster mushrooms) have 34.6 g/100 g TDF, with 30.7 g/100 g IDF and 4.0 g/100 g SDF on a dry weight basis [71]. Dried mushrooms, such as oyster mushrooms (*Pleurotus ostreatus*), exhibit crude fiber contents ranging from 12.58 to 12.59 g/100 g when sun-dried or oven-dried, while dried shiitake mushrooms (*Lentinula edodes*) contain 6.7 g/100 g of TDF, with 6.2 g/100 g as IDF and 0.5 g/100 g as SDF. Drying methods like sun drying and oven drying do not significantly impact the overall dietary fiber content compared to fresh mushrooms. Thus, incorporating both fresh and dried mushrooms into the diet can effectively boost fiber intake and promote overall health [70].

Research on mushroom dehydration and drying methods reveals valuable insights into the fiber content of fresh and dried mushrooms. Studies show that the fiber content of fresh mushrooms is around 12.87 g/100 g, while different drying methods impact the fiber content differently. For instance, cabinet drying was found to retain a higher fiber content of 13.62 g/100 g in dried mushrooms [82]. Additionally, osmotic pretreatments before drying influenced the fiber content, with higher salt concentrations leading to a reduction in fiber content from 12.82 g/100 g to 9.01 g/100 g [18]. These findings highlight the importance of considering drying methods and pretreatments to preserve the fiber content of mushrooms, which are crucial for maintaining their nutritional value throughout the dehydration process.

Chitin, a significant component present in various organisms, including mushrooms, plays a crucial role in their dietary fiber content, making them resistant to digestion [21,83,84,85]. The chitin extracted from mushrooms exhibits self-healing properties when formed into fibers, forming its unique characteristics and potential applications in various areas [84]. Additionally, the extraction of chitin from mushrooms provides a sustainable source of chitin and chitosan, which can have biomedical and environmental interventions through their supply and utilization [83]. This comprehensive analysis highlights the importance of chitin and dietary fibers in mushrooms, which make them nutritious but also more difficult to fully digest for the human body.

### 4.4. Vitamins and Minerals

Research on the nutritional aspects of fresh and dried mushrooms with various drying methods has revealed significant findings. Studies have shown that drying processes impact the nutritional composition of mushrooms, with vacuum freeze drying enhancing the overall nutrient content and consumer preference, while natural air drying increases the volatile flavor compounds [86]. Additionally, UV-irradiated, dried mushrooms retain high levels of vitamin D2 after rehydration and cooking, making them a valuable source of this essential vitamin [72].

The most commonly consumed mushrooms, such as white button mushrooms (*Agaricus bisporus*), oyster mushrooms (*Pleurotus ostreatus*), and shiitake mushrooms (*Lentinula edodes*), are frequently studied for their vitamin D content. These species account for a substantial portion of global mushroom consumption, approximately 74% combined [72]. The primary form of vitamin D found in mushrooms is ergocalciferol (vitamin D2). Mushrooms can synthesize this compound when exposed to UV light, making them a valuable source of dietary vitamin D, especially for individuals with limited sun exposure. Wild mushrooms, particularly the golden chanterelle (*Cantharellus cibarius*) and funnel chanterelle (*Cantharellus Tubaeformis*), were reported to contain high levels of ergocalciferol, with values of 12.8 and 29.82 μg/100 g of fresh weight, respectively [65]. However, commercially sold fresh mushrooms such as button mushrooms (*Agaricus bisporus*) and oyster mushrooms (*Pleurotus ostreatus*) typically have less than 1 μg/100 g of vitamin D2 because they are usually cultivated in the dark, without UV exposure [72].

A study analyzing 35 species of dried mushrooms sold in China reported an average vitamin D2 content of 16.9 μg/g on a dry weight basis, with values ranging from 7 to 25 μg/g on a dry weight basis. Golden chanterelles (*Cantharellus cibarius*) dried using hot air showed vitamin D2 concentrations of between 0.12 and 6.3 μg/g dry matter after being stored in darkness for 2 to 6 years. For button mushrooms (*Agaricus bisporus*), oyster mushrooms (*Pleurotus ostreatus*), and shiitake mushrooms (*Lentinula edodes*), optimal drying conditions were found to be at 60 °C post-UV-B exposure, yielding up to 25 μg/100 g of vitamin D2 on a dry weight basis under ideal conditions [72].

Freeze drying typically maintains a greater quantity of vitamins in comparison to alternative drying techniques. Research suggests that freeze-dried mushrooms hold a higher level of vitamin D2, largely because of the minimized thermal degradation that occurs during the process [7]. Microwave drying is an efficient method for rapidly decreasing moisture levels; however, it may result in the degradation of certain heat-sensitive vitamins. Nevertheless, recent research indicates that employing microwave-assisted freezing before the drying process can reduce ice crystal formation, which may improve the retention of nutrients overall [87,88]. Cabinet drying is recommended as an optimal method for preserving the nutritional components of mushrooms, such as macronutrient minerals, and vitamins B3 and B6, while also improving shelf life and yield [89].

Freeze drying generally results in the highest retention of minerals such as iron, calcium, magnesium, and potassium. For instance, freeze-dried oyster mushrooms exhibited an iron content of 5.10 mg/100 g on a dry weight basis and a calcium content of 16.12 mg/100 g on a dry weight basis; these values were higher compared to when using other methods [29,73]. Hot air drying generally leads to the moderate retention of minerals compared to other methods like freeze drying [2,73]. Specific mineral contents, such as calcium, magnesium, and potassium, may be better retained at lower temperatures of around 30 to 50 °C compared to higher temperatures from 60 to 70 °C, which can cause more significant degradation [90].

Microwave drying has been associated with greater mineral loss compared to freeze drying and even oven drying. This can be attributed to the formation of stable compounds that may bind minerals, making them less bioavailable [73].

Overall, the choice of drying method significantly influences the vitamin and mineral contents of mushrooms, highlighting the importance of selecting appropriate techniques to maximize their nutritional value [7,18,20,68,69,70,71,72,91].

### 4.5. Polyphenols and Antioxidant Compounds

Dehydration techniques significantly influence the retention of the polyphenols and antioxidant compounds in mushrooms. Various methods, including sun drying, hot air drying, microwave drying, and freeze drying, exhibit distinct effects on these bioactive compounds. For instance, studies have shown that freeze-dried mushrooms exhibit enhanced total phenolic content and antioxidant capacity when compared to those dried using hot air or microwave methods [92,93]. The freeze-drying technique is known to maintain the highest concentrations of both free and bound phenolics in mushrooms. Research on *Pleurotus eryngii* indicates that freeze-dried samples possess a greater phenolic content compared to those subjected to other dehydration treatments. Additionally, studies on Romanian wild edible mushrooms like *Boletus edulis* and *Macrolepiota procera* indicated that freeze-dried samples had significantly higher total phenolic content and antioxidant activity compared to their sun-dried counterparts [94]. Furthermore, the combination of hot air and microwave drying techniques has been shown to enhance the drying efficiency and the retention of bioactive compounds when compared to conventional hot air drying alone [95].

Sun drying generally results in the lower retention of polyphenols due to prolonged exposure to sunlight and higher temperatures, which can affect sensitive compounds. While the hot air drying method is effective for moisture removal, it often leads to moderate losses in polyphenolic content and antioxidative activity, due to the heat applied during the process. Conversely, microwave drying has been shown to retain higher levels of polyphenols compared to hot air drying, which is likely due to shorter processing times and lower thermal degradation [96,97].

The antioxidant activity of mushrooms is closely linked to their polyphenolic content. Freeze drying has been shown to maintain or even enhance the antioxidant properties of mushrooms. In particular, one study reported that freeze-dried mushrooms had significantly higher antioxidant activities as measured through various assays (e.g., DPPH radical scavenging), compared to those dried by other methods [98,99].

## 5. Organoleptic Changes in Dried Mushrooms

Dehydration can significantly affect the organoleptic properties of mushrooms, which are crucial for consumer acceptance and overall quality. According to the literature, the drying method significantly influences the color, texture, aroma, and flavor of dried mushrooms; thus, careful control of the drying process is required to optimize the sensory qualities [100,101]. A summary of the nutritional and organoleptic changes in mushrooms is presented in Table 2.

### 5.1. Aroma and Flavor

Various research articles provide detailed insights into the organoleptic characteristics, specifically the aroma and flavor, of dried mushrooms when different drying methods are employed. For instance, studies on the caterpillar mushroom (*Cordyceps militaris*) reveal that vacuum freeze drying (VFD) is particularly effective in preserving volatile and characteristic aroma compounds. This method ensures that the unique scents and flavors of the mushrooms remain intact. Conversely, intermittent microwave treatment combined with hot air drying (MW-HAD) enhances the taste and increases the concentration of bitter-tasting amino acids, providing a richer and more complex flavor profile [54].

Research on the radish-like oudemansiella (*Oudemansiella raphanipes*) further highlights the advantages of various drying methods. Vacuum freeze drying is highlighted for its ability to maintain the appearance and flavor components of dried mushrooms in a state closest to those of fresh mushrooms. This method is particularly adept at preserving the natural esthetics and taste. In contrast, ultrasound-assisted hot air drying at 60 °C retains high levels of free amino acids, which are crucial for an enhanced flavor, ensuring that the dried mushrooms possess a strong and savory taste [86].

Moreover, studies focusing on shiitake mushrooms (*Lentinula edodes*) illustrate the impact of different drying techniques on flavor and sensory quality. Heat pump dehumidifier drying (HPD) has significantly improved the flavor and overall sensory quality of shiitake mushrooms compared to hot air drying and vacuum freeze drying. Notably, HPD drying also boasts lower energy consumption, making it a more efficient and sustainable option [102].

Collectively, these studies underscore the substantial influence that drying methods have on the aroma and flavor profiles of dried mushrooms. They emphasize the critical importance of selecting appropriate drying techniques to not only preserve but also enhance these organoleptic characteristics, ensuring that the dried mushrooms deliver the desired sensory experience.

### 5.2. Color

Research on mushroom color changes when using different dehydration methods reveals significant insights. Belyaeva et al. proposed optimal drying parameters to enhance organoleptic properties and meet quality standards, finding that specific temperature and humidity levels are crucial for maintaining color integrity in dried mushrooms [22]. Adhikari et al. compared the different drying methods and pretreatments, highlighting their impact on color retention, with cabinet drying showing superior results for color preservation due to the controlled environment and gradual drying process, which minimize enzymatic browning and pigment degradation [18].

Popa et al. investigated the effects of drying methods on color changes, noting that centrifugal vacuum drying and freeze drying maintain better color stability compared to hot air drying. This is attributed to the lower temperatures and reduced oxidation in vacuum and freeze drying, which help preserve the natural pigments and reduce the Maillard reaction responsible for browning [43]. This research focused on color changes during drying, emphasizing the darkening effect on *Agaricus bisporus* (button mushrooms), where the high temperatures used in methods like hot air drying accelerate enzymatic reactions and non-enzymatic browning, leading to significant color changes.

Duan and Xu’s study of shiitake mushrooms (*Lentinula edodes*) demonstrates that freeze drying and natural air drying result in the least color alteration, while microwave and infrared drying significantly influence color changes due to the intense heat and rapid moisture removal, causing pigment breakdown and browning reactions [73]. These findings collectively emphasize the importance of choosing appropriate drying methods to preserve the visual and sensory quality of dried mushrooms, which is critical for consumer acceptance and marketability [61,103].

### 5.3. Texture

Studies have shown that the choice of drying method significantly impacts the texture of dried mushrooms. For instance, freeze drying and far-infrared radiation drying have been found to result in dried shiitake mushroom (*Lentinula edodes*) with a better appearance, lower hardness levels, and a higher rehydration ratio compared to other methods like heat pump drying and hot air drying. Freeze drying maintains the original cellular structure due to the sublimation process, leading to a lighter and more porous texture. Using infrared radiation ensures uniform heating and efficient moisture removal, preserving the mushroom’s structure and enhancing textural quality. Heat pump drying and hot air drying, on the other hand, often lead to increased hardness and lower rehydration ratios. Heat pump drying, which uses a refrigeration cycle to dehumidify and heat the air, can cause uneven drying and shrinkage in the mushrooms, resulting in a denser and tougher texture. Hot air drying, a conventional method involving circulating hot air, can cause significant shrinkage and a more rigid texture due to prolonged exposure to heat, which can damage the cellular structure and reduce the moisture content unevenly [54].

Additionally, the use of combined humid-convective drying has been shown to influence the texture of shiitake mushrooms (*Lentinula edodes*), along with their surface color, microstructure, volatile compounds, and polysaccharides. Combined humid-convective drying, which integrates both humid and convective drying techniques, helps in retaining more moisture and preserving the microstructure, leading to a more desirable texture that is less brittle and is more similar to that of fresh mushrooms [104].

These studies highlight the importance of selecting an appropriate drying method to preserve the desired organoleptic characteristics, particularly texture, of dried mushrooms. The method chosen can affect not only the texture but also other sensory attributes such as appearance, color, and aroma, emphasizing the need for careful consideration in the drying process to achieve high-quality dried mushroom products.

**Table 2:** Major nutritional and organoleptic changes when using different dehydration techniques.

Drying Systems	Mushroom Species	Nutritional Changes	Organoleptic Changes	References
Hot air drying	Button mushroom (*Agaricus bisporus*) Shiitake mushroom (*Lentinula edodes*)	Slight reduction in protein content Minimal changes in fibers and minerals Reduced sugar content Moderate loss of vitamins (e.g., vitamin B, vitamin C) Increased polyphenol content and antioxidant activity (aging dried mushrooms)	Increased hardness and chewiness Decreased cohesiveness and springiness Decreased whiteness index Increased yellowness index	[2,105]
Freeze drying	Button mushroom (*Agaricus bisporus*) Shiitake mushroom (*Lentinula edodes*)	Slight reduction in protein content Moderate loss of vitamins High retention of bioactive compounds Higher levels of phenolic compounds Higher levels of antioxidant activity	Decreased hardness and chewiness Porous and spongy texture Retained cohesiveness and springiness Preserved whiteness	[7,28,106]
Oven drying	Button mushroom (*Agaricus bisporus*) Shiitake mushroom (*Lentinula edodes*) Oyster mushroom (*Pleurotus* spp.)	Moderate reduction in protein content Minimal changes in minerals High loss of heat-sensitive vitamins Lower levels of bioactive compounds (phenolic and organic acids in mushrooms) Lower levels of antioxidant activity	Increased hardness and chewiness Increased hardness at higher temperatures Decreased cohesiveness and springiness Decreased whiteness index Increased yellowness index	[22,55,105,107]
Microwave drying	Button mushroom (*Agaricus bisporus*	High retention of proteins compared to hot air drying Minimal loss of vitamins compared to oven drying Higher levels of phenolic compounds Higher levels of antioxidant activity	Decreased hardness and chewiness Porous and spongy texture Retained cohesiveness and springiness Preserved whiteness	[27,107]
Sun drying	Shiitake mushroom (*Lentinula edodes*) Oyster mushroom (*Pleurotus* spp.)	Slight reduction in protein content Better retention of fiber content and crude fiber content Higher levels of vitamins (e.g., vitamin D2)	Increased hardness and chewiness Decreased cohesiveness and springiness Darkening of color	[43,105,108]

The above table summarizes the key changes in both nutritional content and organoleptic properties (such as texture, color, flavor, and aroma) that occur in different mushroom species when subjected to various dehydration methods. The table compares the effects of sun drying, hot air drying, microwave drying, oven drying, and freeze drying on essential nutrients (vitamins, minerals, and bioactive compounds) and how these methods influence the sensory characteristics of the final dried product.

## 6. Microbiological Aspects of Dehydration

Dried mushrooms, which are commonly consumed worldwide, present microbial safety concerns despite being perceived as low-risk foods [109]; various drying techniques significantly impact the microbiological characteristics of dried mushrooms. Studies have shown that drying processes like freeze drying, hot air drying, and centrifugal vacuum drying influence the microbial density and quality of mushrooms [110]. Pretreatments such as UV exposure and the use of ascorbic acid and sodium metabisulfites have been found to effectively reduce microbial contamination during the drying process, enhancing the microbiological safety of dried mushrooms [111].

### 6.1. Microbial Load Reduction

Research has shown that hot air drying can decrease microbial growth in stored mushroom slices due to low water activity, with negligible growth observed after three weeks of storage [62]. Additionally, studies on edible bolete mushrooms (*Boletus* spp.) have shown that hot air drying at different temperatures and durations does not significantly affect their bacterial counts, even after six months of storage at room temperature [111]. Furthermore, water activity (aw) is a critical parameter in the storage and preservation of dried mushrooms; a water activity level below 0.6 is generally recommended for the safe storage of dried foods, including mushrooms. At this level, the growth of bacteria, yeasts, and molds is significantly inhibited, thereby extending shelf life and ensuring safety [90].

These findings suggest that hot air drying methods can effectively preserve dried mushrooms by reducing microbial activity, thus enhancing their shelf life and safety for consumption.

The sun drying of mushrooms has been extensively studied for its impact on the microbiological characteristics of dried mushrooms. Research has shown that sun drying effectively reduces the microbial density of mushrooms, thereby extending their storage life. However, in other studies in the literature, open sun-drying systems generally result in the highest microbial load on the dried mushrooms compared to other drying methods.

Freeze drying has a significant impact on the microbiological characteristics of dried mushrooms. Studies have shown that freeze-dried mushrooms exhibit lower microbial growth due to their low water activity, preserving the mushrooms for an extended period [62]. Furthermore, freeze drying preserves the structural integrity of mushrooms, resulting in less deformation and shrinkage compared to other drying methods, which can lead to microbial contamination [112]. Overall, freeze drying remains a favorable method for preserving the microbiological characteristics and nutritional value of dried mushrooms [60].

### 6.2. Microbial Contamination of Dried Mushrooms

Dried mushrooms can harbor various microorganisms, including aerobic mesophiles, *Enterobacteriaceae*, yeasts, molds, and *Bacillus cereus*. Research indicates that although the average contamination levels are typically low, there are notable variations based on the sample and source. In a recent study examining the microbiological quality of dried mushrooms available for retail, *Bacillus cereus* was detected in 59% of samples at low contamination levels (1.0 to 3.4 log cfu/g), and none of the isolates carried the emetic toxin gene, *ces*. Importantly, the study found no presence of *Salmonella* spp. or *Listeria monocytogenes* in any of the samples [109].

The common contaminants found in dried mushrooms can pose potential risks if not properly handled. Aerobic mesophiles and *Enterobacteriaceae* can indicate poor hygiene or improper handling during processing. Yeasts and molds, while not all harmful, can produce mycotoxins that can be a health concern if consumed [109,113]. *Bacillus cereus*, a spore-forming bacterium, can also survive the drying process and can potentially cause foodborne illness, although the levels found in dried mushrooms were generally low [109].

These findings suggest that while dried mushrooms may harbor *Bacillus cereus*, they are still susceptible to contamination by pathogens such as *Salmonella* and *Listeria*. However, the absence of these pathogens indicates that their overall microbiological quality is generally acceptable. Nonetheless, it is essential to follow appropriate food safety practices, particularly thorough cooking, to mitigate the potential health risks associated with *Bacillus cereus* and other pathogens.

These findings suggest that while dried mushrooms may harbor *Bacillus cereus*, the absence of other common pathogens like *Salmonella* and *Listeria* indicates generally acceptable microbiological quality. Nonetheless, appropriate food safety practices, particularly proper cooking, remain crucial to mitigate the potential health risks associated with *Bacillus cereus*.

## 7. Factors Influencing Nutritional and Microbial Characteristics

The nutritional and microbial characteristics of mushrooms are significantly influenced by various factors during their growth, processing, and storage. Factors such as temperature, moisture content, processing time, and pretreatment methods play crucial roles in determining the quality and safety of the final product. Understanding the impact of these variables is essential for optimizing mushroom-processing techniques to enhance their nutritional value and ensure microbial safety.

### 7.1. Temperature

The drying temperature significantly influences the nutritional and microbial characteristics of dried mushrooms. A higher drying temperature of 70 °C results in greater losses of total phenolic compounds, phenolic acids, organic acids, and ergosterol content compared to a lower temperature of 40 °C in mushrooms like oyster mushrooms (*Pleurotus ostreatus*) and king oyster mushrooms (*Pleurotus eryngii*), which might reduce their health benefits [114,115]. Drying also causes changes in the elemental composition of mushrooms, likely due to the variety of species; the extent of these changes depends on the drying temperature [85]. As reported by Xu et al. in a study investigating the impact of different pre-drying temperatures on the quality of dried shiitake mushrooms, the study found that high-temperature pre-drying at 100 °C for 30 min significantly increased the rehydration ratio and hardness of the mushrooms while reducing shrinkage, browning, and formaldehyde content. Additionally, the process enhanced the enzyme activity, flavor formation, and sulfur compound content, ultimately improving the sensory and nutritional quality of the dried mushrooms.

While drying is an effective preservation method for mushrooms, the drying temperature has a significant impact on their nutritional profile and microbial quality. Lower temperatures of around 40 °C to 50 °C help retain more bioactive compounds and reduce the microbial load, compared to higher temperatures like 70 °C [114,115].

### 7.2. Moisture Content

The moisture content of dried mushrooms significantly impacts their nutritional composition and microbial quality. As the moisture content decreases in raw mushrooms, the relative concentrations of other nutrients like protein, fat, fiber, and ash increase in the dried product [18,82].

The dehydration process reduces the moisture content of mushrooms, from around 90% in the fresh samples to 7–8% in the dried samples. Furthermore, drying at 50 °C significantly decreases the total microbial counts in *Pleurotus ostreatus* (oyster mushroom) and *Pleurotus eryngii* (king oyster mushroom) compared to fresh mushrooms [115]. Dried mushrooms are regularly contaminated with foodborne pathogens like *Salmonella* spp. and *Bacillus cereus* [116,117]. Adequate drying to low moisture levels (aw < 0.85) is important to prevent microbial growth; however, dried foods are not sterile and can be contaminated again in unhygienic conditions [110]. Drying reduces the moisture content, which concentrates the nutrients but also allows some microbial contaminants to survive. Proper drying to very low moisture levels is critical to maximize nutritional quality and minimize food safety risks in dried mushrooms.

### 7.3. Processing Time

Dried mushrooms can be affected by processing times in terms of their nutritional and microbial characteristics. Longer drying times can lead to a decreased protein content and increased Maillard reaction byproducts like reducing sugars, which can degrade the nutritional value of the final product [2]. Prolonged drying can also permit the survival and growth of bacteria, yeasts, and molds, including foodborne pathogens like *Salmonella* and *Bacillus cereus*, which can persist for months or years in low-moisture dried mushrooms [118]. The rehydration of dried mushrooms can further increase the microbial counts, especially those of coliforms [87]. To maintain quality and safety, it is important to optimize the drying methods and times to minimize nutrient degradation and microbial contamination in dried mushroom products [2].

### 7.4. Pretreatment Methods

Various pretreatment methods are employed to enhance the efficiency and quality of mushroom drying. These methods include thermal pretreatment, such as blanching, and nonthermal pretreatment, like sulfiting and osmotic dehydration. Osmotic dehydration serves as an effective pretreatment method prior to freeze drying; this technique involves the partial removal of water from food products using concentrated osmotic solutions, which can significantly improve the subsequent drying outcomes. Osmotic dehydration helps retain essential nutrients and sensory attributes in fruits. For instance, cranberries pretreated with osmotic solutions exhibited superior anthocyanin content and rehydration ratios compared to those dried without pretreatment [119].

Studies have shown that pretreatments like sulfiting can significantly improve nutrient retention, drying time, and the rehydration properties of mushrooms [18]. Pretreatments like blanching and sulfiting can optimize the dehydration properties of milky white mushrooms (*Calocybe indica*). Sulfited samples showed the best nutrient retention with higher crude protein, fat, and fiber contents compared to samples dried using the cabinet, solar, and sun drying methods. Blanching and sulfiting resulted in the shortest drying times, from 8 to 10 h [18]. Different pretreatment methods affect the moisture content and yield of dried mushrooms. Pretreatments like soaking in potassium metabisulfite, fermented whey, or curds reduced the drying times compared to other treatments for both *Pleurotus ostreatus* (oyster mushroom) and *Agaricus bisporus* (button mushroom) [19].

Pretreatments can impact the color and texture of dried mushrooms. Sodium hypochlorite, sodium metabisulfite, and glycerol were effective in reducing the browning of dried gray oyster mushrooms (*Pleurotus ostreatus*). Glycerol pretreatment significantly improved the texture of rehydrated dried mushrooms, making them similar to fresh mushrooms. Calcium chloride and alum produced firmer-textured dried mushrooms, with calcium being more effective than alum [120]. Soaking in fermented whey or curds and using microwave drying can improve moisture migration and reduce drying times. However, proper hygiene and storage conditions are crucial to minimizing microbial contamination in dried mushrooms [19,121].

## 8. Optimization Strategies for the Dehydration Process

Combining different drying methods can improve the rehydration quality of dried shiitake mushrooms [63]. Studies have highlighted the use of osmotic dehydration coupled with hot air drying as a way to produce dried mushrooms efficiently.

In a study by Hu et al. on combined drying methods to improve the rehydration quality of dried shiitake mushrooms, the authors concluded that combined drying methods, particularly those incorporating freeze drying or instant controlled pressure drop drying, significantly improved the rehydration quality of shiitake mushrooms. These methods optimized factors like hardness, sensory attributes, and porosity. Freeze drying followed by hot air drying at 35 °C or 65 °C showed high rehydration rates that were comparable to those dried by freeze drying. Combining instant controlled pressure drop drying with hot air drying at 35 °C also resulted in a rehydration rate that was similar to freeze drying, this being much higher than with hot air drying alone at 35 °C [63].

In summary, combining drying methods like freeze drying with hot air techniques, using cabinet drying instead of sun drying, and using pretreatments incorporating blanching and sulfiting can help optimize the dehydration and rehydration properties of dried mushrooms. However, the choice of method depends on the specific mushroom variety and desired quality attributes.

## 9. Conclusions and Future Perspectives

This comprehensive review has examined the impact of various dehydration techniques on the nutritional and microbial profiles of dried mushrooms. Our findings highlight the importance of selecting appropriate dehydration methods to preserve the nutritional value and ensure the microbiological safety of dried mushrooms.

Freeze drying excels in preserving nutritional quality and maintaining high levels of proteins, vitamins, and minerals, thus making it an ideal method for nutritional preservation. In contrast, hot air drying and microwave drying, while effective for significantly reducing the microbial load, often compromise the nutritional integrity of the food. This highlights a critical trade-off between ensuring microbial safety and maintaining the nutritional value of dried food products.

Hot air drying is a highly effective method for reducing microbial density and extending the shelf life of various products. This technique ensures a significant decrease in the presence of microorganisms, thereby enhancing microbial safety. While sun drying and microwave drying also contribute to the reduction of microbial load, their effectiveness can vary based on specific conditions such as temperature, duration, and the nature of the product being dried. The varying efficiency of these methods underscores the importance of selecting the appropriate drying technique and conditions to achieve optimal microbial safety and product longevity.

Freeze drying is superior in terms of maintaining color stability and texture compared to hot air drying and microwave drying, preserving the organoleptic characteristics of food products more effectively. Freeze drying excels in retaining the original color and texture, making it ideal for high-quality preservation. Freeze drying and cabinet drying are highly recommended for preserving the nutritional components of mushrooms, such as proteins, fats, carbohydrates, minerals, and vitamins B3 and B6, ensuring that their nutritional value remains intact. Additionally, for maintaining the natural aroma and flavor compounds of mushrooms, vacuum freeze drying, along with intermittent microwave treatment combined with hot air drying, has proven to be particularly effective. These methods not only enhance the shelf life and safety of mushrooms but also ensure that their nutritional quality, flavor, and aroma are well-preserved, making them superior choices for the mushroom-drying process.

For the optimal preservation of mushrooms, freeze drying is the preferred method for maintaining nutritional quality and bioactive compounds. Hot air drying is a suitable option for reducing microbial load and extending shelf life. Sun drying can also be utilized for reducing microbial density and extending shelf life, although its effectiveness may vary. For quick drying while preserving quality attributes such as texture and flavor, microwave-assisted hot-air drying is recommended. This has the notable advantage of combining speed and quality retention, especially in the case of mushrooms. This method not only accelerates the drying process but also ensures that essential quality attributes, such as texture and flavor, are preserved, making it a valuable technique for efficient yet high-quality drying.

In the future, advancing the field of mushroom dehydration requires several key steps. Firstly, conducting more rigorous comparative studies is essential to comprehensively grasp how various dehydration techniques impact the nutritional and microbial profiles of dried mushrooms. Additionally, there is a critical need for further research aimed at optimizing dehydration methods to produce dried mushrooms of exceptional quality that are ideal for both culinary and medicinal applications. Moreover, standardizing dehydration techniques across the industry is imperative to maintain consistent levels of quality and safety in dried mushroom products, ensuring reliability for consumers and manufacturers alike. Given the continuous demand for high-quality dried mushrooms and the limitations of existing drying techniques, it is recommended that future research should focus on exploring and optimizing innovative drying methods. In particular, the supercritical drying method has emerged as a promising area of investigation. 

## Figures and Tables

**Table 1:** Effects of various drying techniques on the nutritional composition of different mushroom species.

Nutrient	Mushroom Species	Fresh Mushrooms (Wet Weight)	Fresh Mushrooms (Dry Weight)	Dried Mushrooms (Dry Weight)	Treatment	Literature
Proteins (g/100 g)	Shiitake Mushroom (*Lentinus edodes*)	1.6–2.2	14.87–27.13	24.7	Freeze drying	[54,66]
				15.1–19.1	Different methods	[67]
	Milky White Mushrooms (*Calocybe indica*)		26.60–39.84	22.85	Cabinet drying (60 °C)	[20]
				21.52	Solar drying (40 °C)	[18]
				20.73	Sun drying (27 °C)	
	Oyster Mushroom (*Pleurotus* spp.)		18–19.15	30.81	Hot air drying (40 °C)	[66,68]
	Button Mushroom (*Agaricus bisporus*)		12.05	24.36	Freeze drying	
Carbohydrates (g/100 g)	Button Mushroom (*Agaricus bisporus*)	2.5–4	7.66	8.1	Hot air drying	[69]
				10.1	Freeze drying	
	Milky White Mushrooms (*Calocybe indica*)		26.92	51.17	Cabinet drying (60 °C)	[18]
				51.24	Solar drying (40 °C)	
				52.56	Sun drying (27 °C)	
	Shiitake Mushroom (*Lentinula edodes*)		26.67	7.6–11.4	Different methods	[69]
Fibers TDF (g/100 g)	Oyster Mushroom (*Pleurotus* spp.)	3.3–36.8 TDF	47.3	12.58–12.59	Sun drying Oven drying	[70]
	King Oyster Mushroom (*Pleurotus eryngii*)		47.3	25–30	Different methods	[71]
	Shiitake Mushroom (*Lentinula edodes*)		17.24	6.7	Cabinet drying	[70]
Vitamin D (μg/100 g)	Button Mushroom (*Agaricus bisporus*)	<1	<1	25.0	Cabinet drying (60 °C) Post UV-B	[72]
	Shiitake Mushroom (*Lentinula edodes*)					
	Oyster Mushroom (*Pleurotus ostreatus*)					
	Golden Chanterelle (*Cantharellus cibarius*)			0.12–6.3	Hot air drying	[72,73]
Minerals						
Iron (mg/100 g)	Button Mushroom (*Agaricus bisporus*)	12.3	17.5	5.10	Freeze drying	[73,74]
				4.12	Microwave drying	
				3.80	Sun drying	
Calcium (mg/100 g)	Button Mushroom (*Agaricus bisporus*)	47	36.0	16.12	Freeze drying	
				5.08	Microwave drying	
				3.09	Sun drying	
Magnesium (mg/100 g)	Button Mushroom (*Agaricus bisporus*)	1.1–1.4	7.00	16.12	Freeze drying	
				15.59	Microwave drying	
				15.06	Sun drying	
Total Polyphenols (mg GAE/g)	Button Mushroom (*Agaricus bisporus*)	4.94–7.66	6.43–7.66	15–30 10–25 25–40 35–50	Sun drying Hot air drying Microwave drying Freeze drying	[2,9,29,75]
	Shiitake Mushroom (*Lentinus edodes*)					
	Oyster Mushroom (*Pleurotus* spp.)					

## Data Availability

No new data were created or analyzed in this study. Data sharing is not applicable to this article.
